# Use of Naringinase to Modify the Sensory Quality of Foods and Increase the Bioavailability of Flavonoids: A Systematic Review

**DOI:** 10.3390/molecules30112376

**Published:** 2025-05-29

**Authors:** Joanna Bodakowska-Boczniewicz, Zbigniew Garncarek

**Affiliations:** Department of Biotechnology and Food Analysis, Faculty of Production Engineering, Wroclaw University of Economics and Business, Komandorska 118/120, 53-345 Wrocław, Poland; joanna.bodakowska-boczniewicz@ue.wroc.pl

**Keywords:** naringinase, deglycosylation, bioavailability, flavonoids, hesperidin, rutin

## Abstract

As a complex of enzymes α-l-rhamnosidase and β-d-glucosidase, naringinase catalyzes the deglycosylation of flavonoids. According to the PRISMA method, a systematic literature review was conducted analyzing peer-reviewed scientific articles from the Scopus and Web of Science databases. Recent reviews on naringinase have focused on its sources, production, and general applications, whereas the present study highlights its specific applications, its role in the deglycosylation of flavonoids, and the resulting improvement in their bioavailability. This review focuses on advances in modifying the glycosidic parts of various flavonoids using naringinase by selectively disconnecting the rhamnose or glucose moiety. Removing rhamnose from the flavonoid molecule but leaving the glucose allows more water-soluble compounds to be present and increases bioavailability. A necessary condition for such selective deglycosylation is removing or inhibiting beta-glucosidase, the second enzyme in the native naringinase molecule. The use of naringinase for preparing functional beverages with increased antioxidant activity and for preparing steviol sweeteners is also presented. Naringinase enables the desired sensory properties to be obtained, primarily the taste and aroma of food products, and can be used in combination with other enzymes, e.g., pectinase and tannase.

## 1. Introduction

Naringinase is an enzyme with dual activity: α-l-rhamnosidase (EC 3.2.1.40) and β-d-glucosidase (EC 3.2.1.21). Naringinase and α-rhamnosidase are often used as synonyms in the literature. Naringinase, hesperidinase, and rhamnosidase have the same EC number (EC 3.2.1.40). Naringinase and hesperidinase are commercial enzymes containing α-l-rhamnosidase and β-d-glucosidase activity [1]. Some authors define naringinase as an enzymatic complex with α-l-rhamnosidase and β-d-glucosidase activity [2]. Still, others claim that naringinase is a mixture of two separate enzymes, α-l-rhamnosidase and β-d-glucosidase [3].

The catalytic mechanism of naringinase is attributable to its membership in the glycoside hydrolase family [3]. The protein structure of α-l-rhamnosidase reveals that the enzyme has a significant role in naringinase functionality and activity and that numerous negatively charged residues, including *Asp568, Glu572, Asp579*, and *Glu841*, potentially play a key role in enzymatic catalysis and substrate binding [4].

Naringinase has been reported in the literature since 1938. It was initially isolated from celery seeds and later from grapefruit leaves [3]; most notably, it has been found in the molds of the *Aspergillus* genus [5], *Penicilium* [6], as well as bacteria [7,8,9,10] and yeast [11]. However, it should be emphasized that, due to the use of naringinase, the enzyme should come from microorganisms included in the GRAS (Generally Recognized As Safe) group.

Several review articles on naringinase have been published in the past ten years. These studies primarily focus on the microbiological sources of the enzyme and generally discuss its applications—mainly the removal of bitterness, but also highlighting its potential medical uses [12,13,14,15]. The data presented in this article provide a more detailed account of the application of naringinase in the juice industry and, more importantly, of its role in the deglycosylation of flavonoids and the resulting enhancement of their bioavailability.

The present study aims to demonstrate the potential of naringinase in the selective deglycosylation of flavonoids by targeting rhamnose or glucose moieties, with a focus on enhancing the bioavailability of the resulting aglycones and glucoside derivatives and exploring its applications in the development of functional beverages and steviol sweeteners, and in improving the sensory properties of food products.

There are only a few commercial manufacturers of naringinase, mainly from China. Although many microorganisms can synthesize naringinase, most commercially available products are of fungal origin. An obstacle to obtaining fungal naringinase is the slow growth rate of naringinase-producing fungi. UV-ARTP combinatorial mutagenesis was recently used to obtain an *A. tubingensis* strain with higher productivity and naringinase activity [16]. The highest naringinase activity of 2475 ± 16 U mg^−1^ was obtained after only 96 h of culture. These results show that naringinase production can significantly increase using combined ARTP (Atmospheric- and Room-Temperature Plasma) and UV mutagenesis. In addition, a new and simple screening method for naringinase-producing microorganisms was developed based on their growth in the presence of naringin as the sole carbon source, followed by plate exposure to iodine vapor [17]. This method can potentially fill the gap left by the unavailability of an effective screening methodology for industrially important naringinase-synthesizing microorganisms.

Many natural glycosides containing an α-rhamnose or β-glucose residue can be substrates for naringinase. These include naringin, rutin, quercetin, hesperidin, neohesperidin, diosmin, myricitrin, monoterpenes, and some saponins, including ginsenosides [1,6,18].

## 2. Methodology

This study followed the PRISMA (Preferred Reporting Items for Systematic Reviews and Meta-Analyses) guidelines to conduct the systematic review study [19]. This study employed two electronic databases (Scopus and Web of Science) and used the keyword “naringinase”. Boolean operators were not used. The search and selection process was performed by two reviewers working independently and in parallel.

The study for this systematic review was restricted to articles published between 2005 and 2025. For databases, four primally exclusion steps were determined: (1) keyword (“naringinase”); (2) year; (3) language (English); (4) publication type (article). Screening was performed in the following areas: Title, Abstract, Keywords. The last search was carried out on 27 February 2025.

Among the initial 578 reports collected through the search, 201 were omitted due to duplicate results, from which 131 were removed because of the year and 39 were removed because of the study type. Also, 27 were excluded because they were not in English language. Of 180 retrieved reports, 115 articles were deemed irrelevant based on abstract and/or title information.

**Figure 1:**
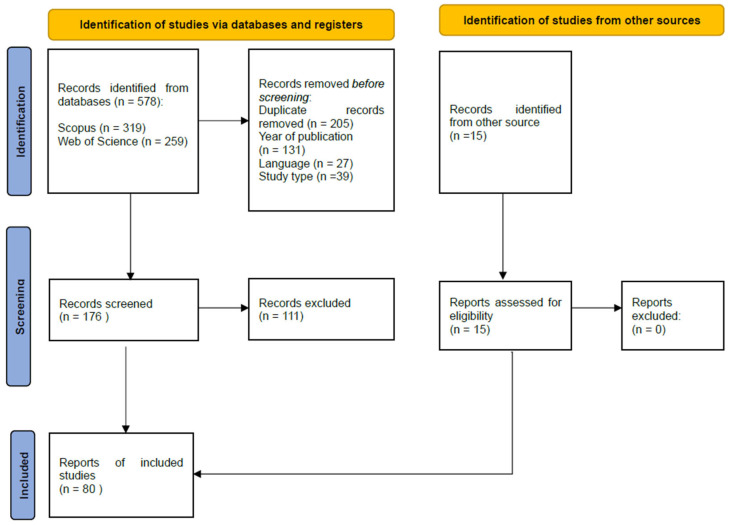
The PRISMA flow chart of the selection process for the included studies.

Before conducting the systematic literature search following the PRISMA methodology, the authors had previously identified 15 articles based on prior research, expert recommendations, and manual screening of the literature. The review included these articles as additional sources (“Identification of studies from other sources”). They were subjected to the selection process according to the predefined inclusion and exclusion criteria. Finally, 80 articles were included in this study, as demonstrated in a flowchart of the literature search and selection process (Figure 1). The Prisma checklist was added as Supplementary Material.

## 3. Use of Naringinase

Naringinase is an enzyme complex used in the deglycosylation of compounds with high application potential in the food industry. Naringinase is used in the alcoholic beverage industry (wine and beer) and the soft drink industry (teas and juices). The beverage industry is constantly looking for innovations that improve fruit and floral flavors to increase the diversity of existing flavor profiles [20].

This enzyme is critical in citrus processing, where hydrolyzing naringin can remove bitterness. It is also used to improve the flavor of wines, juices, and other beverages [5,21]; it can increase the bioavailability of flavonoids and antioxidant potential, can be used to produce sweeteners, and can improve the quality of soy products.

### 3.1. Removing the Bitter Taste of Citrus Juice

The bitter taste of some citrus species, especially grapefruit, is considered a desirable quality trait [22], but only if it is not in excess. Naringin, a flavonoid glycoside, and the limonoids limonin and nomilin are primarily responsible for the bitter taste of citrus fruits [6].

Adsorption techniques [23] or enzymatic hydrolysis [4,10] are used to remove the bitter components of citrus juices. Today, consumers expect maximum preservation of fruit products’ endogenous sensory, nutritional, and health properties. The use of new enzyme technologies, which have little effect on altering the organoleptic and health characteristics of citrus fruits, can be considered as an alternative to the conventional bitter taste removal process, physical adsorption [24]. Fresh grapefruit juice is the richest source of naringin. The content of naringin in grapefruit juice is about 230–840 µg cm^−3^ [7,24]. High concentrations of naringin are also found in the juice of pomelo, bitter orange, or kinnow mandarins.

**Figure 2:**
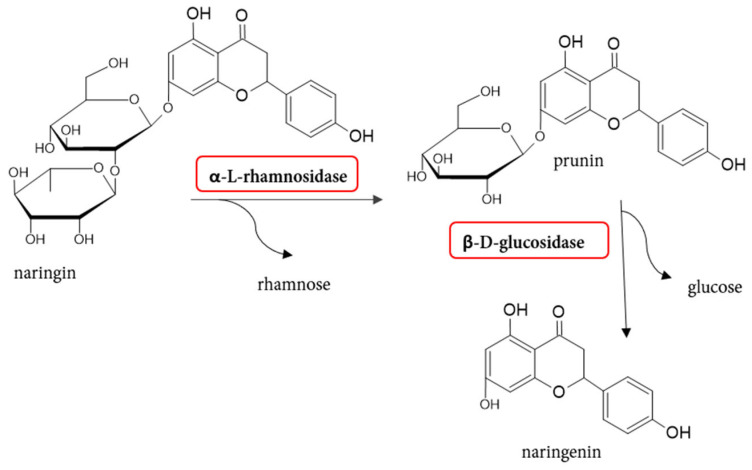
Reaction scheme of the naringin hydrolysis catalyzed by naringinase.

The specific reaction of naringinase is the hydrolysis of naringin. The naringin molecule contains the aglycone naringenin and two sugar moieties, a rhamnosidic and a glucosidic residue, which are linked by an α-1,2 glycosidic bond [3]. Two enzymes, α-l-rhamnosidase and β-d-glucosidase, are required for the complete deglycosylation of naringin. The first of these, α-l-rhamnosidase, hydrolyzes naringin to rhamnose and prunin. Next, β-d-glucosidase hydrolyzes prunin to glucose and naringenin (Figure 2).

The reduction in bitter taste is directly related to the reduction in naringin. It is reported that the bitterness of prunin is already almost 70% less than that of naringin. Further hydrolysis of prunin by β-d-glucosidase leads to tasteless naringenin [22]. Many publications have been devoted to the enzymatic removal of the bitter taste from citrus juices through a naringinase preparation. Table 1 summarizes studies on this topic.

Various forms of naringinase have been used to remove bitterness enzymatically. The results of ongoing studies indicate the potential for enzymatic removal of bitterness from citrus juice and the possibility of mutagenesis or overexpression of genes to increase naringinase activity.

In research on bitter taste removal, attention should be paid to temperature in the processing. Temperature is important in preserving fruit products’ endogenous sensory, nutritional, and health properties. A low temperature in the technological process preserves the sensory properties of the juice. At such temperatures, with short exposure times, the shelf life of thermolabile components of citrus juices is more significant, which does not cause tangible changes in the sensory qualities of products. Most authors have used elevated temperatures around 50° C for the hydrolysis of naringin in citrus juices, which did not leave the sensory qualities of the juices unaffected. Nevertheless, further research on optimizing and scaling up the process is needed before full commercialization.

Other potential applications of the enzyme are being explored, such as using naringinase to remove bitter taste. Del Nobile et al. [40] obtained an active food film capable of hydrolyzing naringin contained in grapefruit juice during storage. The active film consists of a glutaraldehyde-crosslinked matrix on which naringinase from *P. decumbens* was immobilized.

Naringinase and enzymes that degrade hemicellulose and cellulose were used to improve yuzu powder’s functional and organoleptic properties. Applying such an enzyme system reduced the bitterness of yuzu powder by 50% compared to the control sample. The treatment of yuzu powder with the enzyme combination improves its physicochemical properties and biological activity. It reduces its bitterness, thus expanding its application in the food, nutraceutical, and cosmetic industries [41]. Also, naringinase from *A. oryzae* NYO-2 reduced the content of naringin and neohesperidin in yuzu fruit powder by 87.8% and 89.4% [42].

Using naringinase from *Thermomicrobia* sp. reduced the bitterness of kinnow fruit pomace, which was then used to produce nutrient-rich pasta. Under optimized conditions in the fruit pomace, the naringin content was reduced by 65.95%, combined with an increase in the concentration of naringenin (60.13%). Kinnow pomace is rich in fiber, phytochemicals, and antioxidants. Adding to semolina, pomace, after enzymatic treatment, resulted in pasta rich in nutrients and antioxidants. The resulting pasta had an original flavor and an attractive orange color and had the right consistency. Studies have shown that enzymatically processed kinnow pomace can successfully prepare nutrient-rich pasta and other extruded food products [32].

### 3.2. Flavor Enhancement of Fruit Juices and Wines

Compounds that play an important role in shaping the aroma of wine and juices can exist in free form or as non-volatile, odorless glycosides, so-called aroma precursors. Volatile aglycones are released from the glycosidic forms during production or storage.

Aroma precursors are built from an aglycone and a sugar residue, linked by a β-d-glycosidic bond. Aglycones are directly linked to β-d-glucopyranose, which can also be substituted with a second sugar unit with α-l-arabinofuranose, β-d-apiophuranose, and α-l-ramnopyranose, among others [43]. Flavor compounds can be removed from their glycosidic forms naturally during fruit ripening or by hydrolysis by exogenous enzymes.

Improvements in flavor and aroma often result from the activity of glycolytic enzymes already in the plant, present at fermentation sites, or added during winemaking [44]. For naringinase to work effectively under typical winemaking conditions, it should have a tolerance to low pH (3.2–4.0), activity in high sugar concentrations (up to 20% *w*/*v*), and high ethanol concentrations (10–15% *v*/*v* in wine), as well as activity in the presence of sulfites and other compounds [44]. In order to improve the aroma of juices and wine, many authors have investigated the possibility of enzymatic hydrolysis of aroma precursors by using enzymes such as β-d-glucosidase, α-l-rhamnosidase, β-apiofuranosidase, and α-l-arabinosidase [43].

Some authors have tested the usefulness of naringinase in enhancing the aroma intensity of juices and wines [21,45]. It has been shown that using free naringinase from *P. decumbens* resulted in a 1.5-fold increase in the impression of aroma intensity of freshly squeezed grapefruit juice and *Pinot noir* wine [21].

Ni et al. [46], based on a study combining gas chromatography, mass spectrometry (GC-MS), and sensory evaluation, found that, after adding naringinase from *A. niger* to pomelo juice, aroma sensations more than doubled. In addition, it was described that using naringinase and a pectinolytic enzyme preparation in citrus juice production also improves the yield of the juice process [45].

An α-rhamnosidase from *Pediococcus acidilactici* combined with a bacterial β-glucosidase was used to release grape-derived terpenes in Muscat wine. Under optimal conditions, the enzymes released linalool and cis-linalool oxide from Muscat wine extract. In addition, α-rhamnosidase can release significant amounts of geraniol and citronellol/nerol. However, due to the substantial adverse effects of acidity and ethanol on the activity of these enzymes, their use is limited [47].

In a study by Gao et al. [48], ougan juice was treated with α-l-rhamnosidases, β-glucosidases, and limoninases from *A. niger*. The mixture was subjected to ultrasound treatment at 20, 28, 40, and 68 kHz frequencies for 10 to 120 min at temperatures between 20 °C and 70 °C. This enzymatic and ultrasonic treatment effectively reduced the juice’s green, citrus-like, floral, and woody notes while enhancing fruity and sweet notes by 18% and 15%, respectively. As a result, the overall taste and aroma scores increased by 38% and 33%.

Glycosides bound to volatile molecules are also generally considered precursors of tea aroma. Ni et al. [49] improved the aroma of white tea using β-glucosidase. The use of β-glucosidase and α-rhamnosidase led to an increased ability to release aromatic tea components from *Ginkgo biloba* leaves [50]. Like wine production, exogenous β-glucosidases can be added to the beer production process to optimize the release of glycoside-linked aromatic molecules, thereby improving beer quality [44].

However, their application is limited due to the substantial adverse effects of acidity and ethanol on enzyme activity. Further research is needed on enhancing enzyme stability and activity at low pH and elevated alcohol content.

### 3.3. One-Time Clarification and Removal of the Bitter Taste of Beverages

Choosing a good clarification method is essential to preserve fruit juices’ natural essence, color, rheological properties, and texture. Fresh fruit juices are viscous and cloudy due to the presence of various polysaccharides such as pectin, cellulose, and starch. These polysaccharides lead to the formation of a colloidal stable suspension of insoluble particles, which hinders the clarification process and thus reduces the quality of fruit juice [51]. Therefore, it is essential to decompose these polysaccharides, after which the filtered juice can be stored for further use. Using enzyme blends such as pectinase, tannase, carbohydrase, naringinase, and lipase/esterase can provide better juice yield and help maintain better nutritional values.

In a study by Ladole et al. [51], pectinase and naringinase were immobilized on chitosan-coated magnetic nanoparticles (chitosanMNP). The co-immobilized biocatalysts were evaluated for clarification and bitterness removal from grapefruit juice, and a 52% reduction in turbidity and an 85% reduction in naringin content were found. Naringin hydrolysis is facilitated by reducing juice turbidity due to simultaneous pectinase treatment. By reducing the viscosity of the juice, naringinase can act more effectively.

Kumar et al. [52] successfully removed bitterness and clarified pomelo juice using immobilized enzymes naringinase and tannase. Ni et al. [46] found that combining pectinase increased juice yield and bitterness removal. There was an increase in soluble pectin content, total soluble solids (TSS), and juice clarity, while the bitterness of naringin, limonin, and nomilin decreased.

Other researchers added 4% pectinase enzyme to grapefruit juice and performed a clarification reaction for 90 min at 45 °C. After centrifugation, a clear juice was obtained, which was then treated with a naringinase derived from *Serratia marcescens* to remove naringin. Under optimal conditions, the enzyme hydrolyzed 88.85% of the naringin contained in the clear grapefruit juice [36].

In addition, it has been shown that β-glucosidase can reduce wine coloration. β-glucosidase hydrolyzes the anthocyanins responsible for wine color. The breakdown of anthocyanins produces anthocyanidins, which naturally decompose into colorless compounds [44].

One of the main challenges in using different enzymes is the need to select optimal pH and temperature values common to the activity of all enzymes. Additionally, free naringinase poses several practical limitations, such as the inability to reuse the enzyme, its sensitivity to environmental changes, and difficulties separating the enzyme from reaction mixtures. These issues can be effectively addressed through various enzyme immobilization techniques.

### 3.4. Increasing the Bioavailability of Flavonoids

Flavonoids are usually found in plants as aglycones bound to sugar residues and, in this form, they are supplied to the human body with food [53]. Despite the broad spectrum of therapeutic activities of flavonoids, their use is limited by poor absorption due to their low solubility in water. Poor solubility of flavonoids results in low bioavailability and high variability in their absorption, which limits their use as food additives or dietary supplements [18]. Glycosylation naturally affects flavonoids’ biophysical and biochemical properties and their biological activity [54]. However, the effect is dependent on the respective sugar groupings. While glucosylation or galactosylation usually increase the solubility of flavonoids in water, the presence of rhamnosyl residues slightly reduces it. For example, the 7-*O*-glucoside of hesperetin is about 50 times more soluble in water [55] and 55 times more soluble in 10% ethanol than hesperidin [56]. The presence of sugar groupings can lead to a change in the bioavailability of the corresponding flavonoid aglycone depending on the nature of the sugar, as, for example, glucosides are absorbed faster than other types of glycosides, such as rhamnosides and rhamnoglucosides. Due to the hindered absorption of flavonoids, solutions seek to increase their bioavailability, such as by deglycosylation [54].

The lower bioavailability of some flavonoids is mainly due to the lack of suitable hydrolyzing enzymes in the human gastrointestinal tract. While glucosides can be cleaved by intestinal lactose-floroside hydrolase or β-glucosidase of small intestinal epithelial cells, there is no human α-l-rhamnosidase or rutinosidase, and the bioavailability of rhamnose-containing flavonoids is entirely dependent on their cleavage by the intestinal microbiota [54,57]. Therefore, compared to other flavonoid diglycosides, flavonoid mono glucosides have higher bioavailability and are easier to absorb and metabolize in the human digestive tract [57].

Unlike glucosides, which glucosidases can break down throughout the gastrointestinal tract, rutinosides, including disomine, can only be digested in parts of the gastrointestinal tract where there are enterobacteria capable of producing enzymes with rhamnosidase activity that cleave off rhamnose molecules in the terminal position. Diosmin is converted by gastrointestinal bacteria into aglycone diosmetin during digestion and is absorbed from the gastrointestinal tract in this form. The degree of deglycosylation of flavonoids can be increased by supplying these compounds to the food along with appropriate enzymes. Naringinase can be microencapsulated with rhamnose-containing flavonoids such as diosmin or hesperidin. Microencapsulated naringinase increases the absorption of flavonoids from the gastrointestinal tract by enzymatic breakdown of the flavonoid molecule containing rhamnose to its aglycone. Diosmin is hydrolyzed to diosmethine in the gastrointestinal tract, and hesperidin is hydrolyzed to hesperetin [18].

Several attempts have been made to increase the bioavailability of flavonoids. A patented method for complexing the flavonoids contained in ginkgo biloba extract involved treatment with naringinase in the presence of γ-cyclodextrin. Incorporating of ginkgo biloba extract into γ-cyclodextrin in the presence of naringinase improves the bioavailability of flavonoids, primarily quercetin, kemferol, and isoramnetin [58]. In addition, inclusion complexes of isoquercitrin in cyclodextrin and hesperetin 7-*O*-glucoside (Hes-7-G) in β-cyclodextrin showed about 10- and 100-fold higher bioavailability in humans than their rutinoside forms, respectively. An inclusion complex of diosmethin glucoside in γ-cyclodextrin was prepared by reacting diosmethin and naringinase with γ-cyclodextrin. The bioavailability of diosmetin glucoside was about 800 times higher than that of biosmetin after its administration in rats [59].

Enzymatic hydrolysis via naringinase may be a promising method for increasing the bioavailability of ginsenosides [60]. Ginsenosides are the main active component of ginseng. A practical method has been developed to convert ginsenosides into a highly bioactive compound K, i.e., 20-*O*-β-d-glucopyranosyl-20 (*S*)-protopanaxadiol. Due to the removal of glycosyls, this compound shows high bioactivity and bioavailability after the oral administration of ginseng. Ginsenosides can be efficiently converted to compound K by naringinase. The optimal conditions for enzymatic hydrolysis were a pH of 4.1, a temperature of 50 °C, and a time of 71 h; the reaction yield was 65.44 ± 4.52% [61].

The enhanced bioavailability of flavonoids was also achieved through their esterification. *A. oryzae* cells cultured in the presence of a naringin-containing inducer exhibited both lipase and naringinase activity, enabling two processes to be carried out simultaneously—the hydrolysis of naringin to naringenin and the acylation of naringin to its esters. The hydrolysis catalyzed by naringinase yielded naringenin, which improves sensory quality by reducing the bitter taste. At the same time, acylation catalyzed by lipase yielded naringin esters with different fatty acid chain lengths. The obtained esters showed significantly better antioxidant activity and increased bioavailability than naringin alone. Whole cells of *A. oryzae* can effectively produce lipophilic derivatives of naringin in citrus extracts. These derivatives may find wider applications than naringin in the food and pharmaceutical industries, opening up new prospects for developing the citrus processing industry [62].

#### 3.4.1. Hydrolysis of Naringin

Naringin and its hydrolysis products, rhamnose, prunin, and naringenin, are the starting materials for synthesizing substances used in the pharmaceutical, cosmetic, and food industries. The results of many studies confirm that naringin and its hydrolysis products have antioxidant, anti-inflammatory, anti-ulcer, and anti-cancer properties [37,63,64]. Naringin, prunin, and naringenin have similar uses, so it can be inferred that the biological activity of these flavonoids is related to the aglycone grouping rather than the presence of sugar residues.

Naringinase may effectively increase the potential bioavailability of naringin by converting it into another component, prunin [65]. The solubility of prunin is 5.81 ± 0.12 mM and is 7.6 times higher than that of naringin, suggesting that prunin may be a more bioavailable food compound [64]. The solubility of naringenin is 0.068 ± 0.01 mM, which is ~1/10 that of naringin.

Efficient methods have been developed to produce rhamnose and prunin by inactivating β-d-glucosidase from naringinase. Naringinase in an alkaline environment loses β-d-glucosidase activity and thus can produce only intermediates [66]. Selective inactivation of β-d-glucosidase from naringinase was also achieved at acidic pH, maintaining very high α-l-rhamnosidase activity (78%). It was a key development toward an easy and inexpensive method of producing expensive flavonoids such as prunin [2]. Chang et al. [65] obtained a purified preparation of naringinase from *A. sojae* with high α-l-rhamnosidase activity and low β-d-glucosidase activity. Enzymatic bioconversion of naringin to prunin by naringinase proceeded with 91% efficiency. Kaur et al. [67] hydrolyzing naringin extracted from citrus peel indicated that recombinant α-l-rhamnosidase has industrial applications for producing rhamnose and prunin.

Carceller et al. [63] showed that naringinase covalently immobilized on graphene oxide has a high potential to produce prunin and naringenin. Naringinase from *P. decumbens* was purified, to produce prunin resulting in an enzyme with high α-rhamnosidase activity. Naringinase from *P decumbens*, characterized by high α-l-rhamnosidase activity, was covalently immobilized on silica zeolite carrier ITQ-2 and used to hydrolyze naringenin. This process yielded prunin and naringenin with a conversion rate exceeding 90% and excellent selectivity [68].

Recently, naringenin was obtained through the hydrolysis of naringin by naringinase from *A. oryzae*, which was then combined with nanosilver. The resulting preparation exhibited enhanced antimicrobial potential against protozoa, bacteria, and fungi [69]. Wang et al. [70] developed an artificial naringinase system by jointly immobilizing α-l-rhamnosidase from *A. oryzae* FJ0123 and β-glucosidase from *Thermotoga maritima* MSB8 on magnetic-silica-based chitosan microspheres. The molar ratio of α-l-rhamnosidase and β-glucosidase in the optimal system was 3:1. Using such an enzyme system, a yield of 96% naringenin was obtained within 2 h without prunin accumulation. Artificial fusion naringinases were developed to compensate for natural enzyme defects and improve naringenin production efficiency. Models of three fusion naringinases were developed by direct fusion or peptide linkers and expressed in *E. coli* BL21. It facilitated the efficient hydrolysis of naringin to naringenin, with a final yield of 13.5 mg·cm^−3^ at a time/space efficiency of 2.25 mg·cm^−3^·h^−1^. These results demonstrate the potential of artificial fusion naringinases for efficient bioconversion of naringin to naringenin [71].

The efficiency of naringin hydrolysis to prunin depends on the inactivation of β-d-glucosidase. Deglycosylation of flavonoids solely by α-l-rhamnosidase, a component of naringinase, results in glucoside-containing compounds with potentially higher bioavailability. Therefore, the selective inhibition of β-d-glucosidase from naringinase is crucial for improving the bioavailability of flavonoid glycosides. Achieving high process efficiency requires the development of a method that effectively inactivates β-d-glucosidase while maintaining high α-l-rhamnosidase activity. It may involve optimizing environmental conditions such as pH and temperature or using specific, preferably natural, inhibitors.

#### 3.4.2. Hydrolysis of Hesperidin

**Figure 3:**
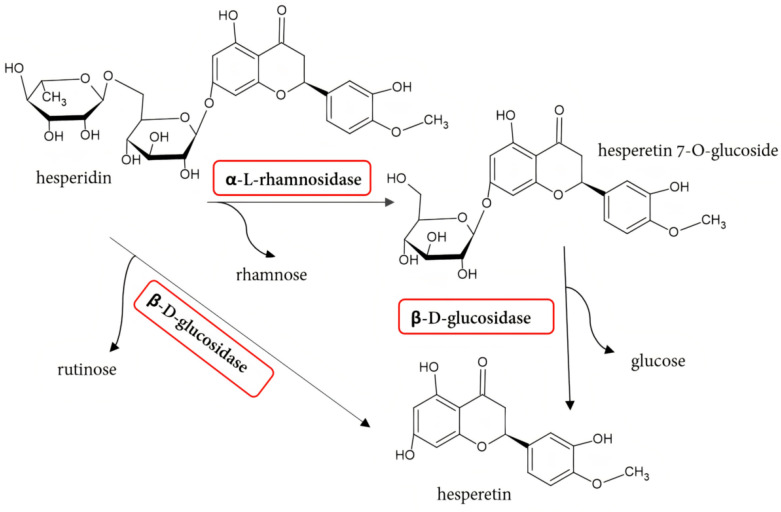
Reaction scheme of the hesperidin hydrolysis catalyzed by naringinase.

Hesperidin is a flavonoid in citrus fruits, primarily oranges, lemons, tangelo, and limes [72]. It is relatively cheap and readily available and can be obtained from industrial orange peel waste, among others [53,72]. It is an excellent starting material for obtaining derivatives with greater antioxidant capacity [56]. Hesperidin contains the aglycone hesperetin and two sugar moieties, a rhamnosidic and a glucosidic moiety, linked by an α-1-6-glycosidic bond. There are two conversion pathways of hesperidin to hesperetin: hydrolysis of hesperidin catalyzed by α-l-rhamnosidase and β-d-glucosidase and direct hydrolysis of hesperidin by β-d-glucosidase (Figure 3).

Hesperidin can be deglycosylated by α-rhamnosidase to rhamnose and hesperetin 7-*O*-glucoside, which β-d-glucosidase then hydrolyzes to glucose and hesperetin [53,56,72]. In the second pathway, β-d-glucosidase catalyzes the cleavage of the glucosidic bond of hesperidin by removing the rutinosylglycoside, directly leading to the formation of hesperetin [54].

Often, only α-l-rhamnosidase is used to hydrolyze hesperidin, obtaining hesperetin 7-*O*-glucoside. As a product of hesperidin derhamnosylation, it is considered a synthetic precursor to new and effective sweeteners [54]. In addition, hesperetin 7-*O*-glucoside inhibits key maltase and cholesterol synthesis enzymes more effectively than hesperidin and its aglycone, thus showing practical anti-diabetic and cholesterol-lowering effects [55,56]. In addition, hesperetin 7-*O*-glucoside more effectively inhibits the growth of *Helicobacter pylori* [56]. It was also found to exert hypotensive and vasodilatory effects [55]. The results of a study on the hydrolysis of hesperidin in orange juice and seeds by naringinase from *A. sojae* indicate that it is more soluble than hesperidin, which affects its higher bioavailability [14,56]. The increased solubility of hesperidin and hesperetin is desirable for practical applications in the food industry [56].

Adding appropriate sugars to the hesperidin hydrolysis reaction environment can effectively achieve the controlled production of hesperetin 7-*O*-glucoside or hesperetin. Rhamnose inhibits the first step of hesperidin conversion and cuts off the α-l-rhamnosidase/β-d-glucosidase pathway, thus promoting the direct hydrolysis of hesperidin to hesperetin. Disaccharides (such as maltose and sucrose) and starch-containing glucosidic bonds also affected the production of hesperetin 7-*O*-glucoside and hesperetin [53]. Many authors have successfully hydrolyzed hesperidin using naringinase, as well as α-l-rhamnosidase and β-d-glucosidase (Table 2).

β-glucosidase from *Pyrococcus furiosus* was used to hydrolyze the flavonoid glycosides hesperidin, neohesperidin, naringin, and narirutin obtained from the citrus extracts of grapefruit seed, grapefruit pulp, and orange seed; β-glucosidase hydrolyzed the flavanone glycosides into their aglycones and disaccharides in a one-step reaction. β-glucosidase completely converted hesperidin in the orange peel extract to hesperetin within 9 h of reaction, yielding 1 g dm^−3^ h^−1^. The enzyme also efficiently hydrolyzed naringin and narirutin contained in grapefruit peel and flesh extract to naringenin. β-glucosidase from *Pyrococcus furiosus* may be helpful for the industrial hydrolysis of flavanone glycosides in citrus extracts [73].

Lee et al. [56] carried out the hydrolysis of hesperidin using naringinase from *A. sojae*, which has high α-l-rhamnosidase activity and relatively low β-d-glucosidase activity, to convert it into the intermediate product hesperetin 7-*O*-glucoside. Hesperidin in orange juice and peels was efficiently converted by α-l-rhamnosidase from naringinase to glucoside, producing a negligible amount of the aglycone hesperetin. The efficiency of hesperetin 7-*O*-glucoside production, relative to the amount of hesperidin found in the extracts, was estimated to be about 71% for orange juice and 78% for orange peel [56].

Kapoor et al. [74] obtained an inclusion complex of hesperetin 7-*O*-glucoside with β-cyclodextrin by enzymatic hydrolysis of hesperidin with naringinase in the presence of β-cyclodextrin. For this purpose, hesperidin and β-cyclodextrin were mixed in water at 70 °C, and the pH was adjusted to 4.5, followed by naringinase hydrolysis for 24 h. Under optimal conditions, the enzymatic conversion of hesperidin to hesperetin 7-*O*-glucoside occurred with an efficiency close to 98%. The results showed that the water solubility and thermal stability of hesperetin 7-*O*-glucoside were enhanced in the inclusion complex with β-cyclodextrin. It could lead to increased bioavailability of this glycoside [74].

Similarly to naringin hydrolysis, the production of hesperetin glucoside requires specific inactivation of β-d-glucosidase while maintaining high α-l-rhamnosidase activity.

#### 3.4.3. Hydrolysis of Rutin

**Figure 4:**
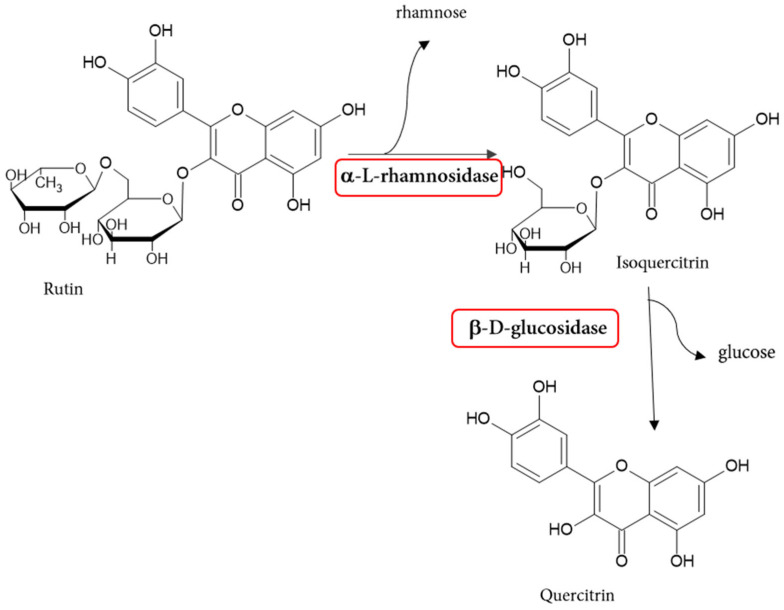
Reaction scheme of the rutin hydrolysis catalyzed by naringinase.

Rutin, hesperidin, and naringin are among the most common glycosylated flavonoids in buckwheat, apples, grapes, tomatoes, and citrus fruits [54]. Rutin contains a disaccharide grouping: α-l-ramnosyl-β-d-glucoside bonded to the flavonoid aglycone at position 3. Rutin, like hesperidin, can be readily available in large quantities and is of high quality at affordable prices, making it an ideal starting material for obtaining more valuable flavonoids, mainly isoquercitrin [54].

a-l-rhamnosidase from naringinase hydrolyzes rutin (quercetin 3-*O*-rutinoside) to rhamnose and quercetin 3-*O*-glucoside (quercetin-3-*O*-glucoside Q-3-G or isoquercitrin) [2,75,76]. β-d-glucosidase, in turn, hydrolyzes isoquercitrin to glucose and quercitrin (Figure 4).

Quercetin and isoquercitrin are compounds with a wide range of biological activity [75]. Quercetin, when administered orally, is poorly absorbed in the gastrointestinal tract and is reported to have an absorption rate of less than 1% [77].

Isoquercetin exhibits anti-inflammatory, antioxidant, and antiproliferative properties and protects against atherosclerosis. In addition, isoquercetin is the primary synthetic precursor of enzyme-modified isoquercetin (EMIQ), derived from rutin produced by transglycosylation with cyclodextrin glucanotransferase. EMIQ isoquercetin, a commercial water-soluble flavonol glycoside, has been approved as a multicomponent food additive [78].

Table 3 summarizes studies on the hydrolysis of rutin by naringinase and its subunits.

Naringinase from *P. decumbens* was used to hydrolyze rutin to aglycone. Quercetin was obtained from rutin with 86% yield [2]. Naringinase has also been used to hydrolyze the rutin contained in buckwheat (*Fagopyrum tataricum*), leading to the formation of quercetin [81].

β-d-glucosidase activity is not desirable for obtaining monoglycosylated flavonoids via naringinase. It leads to the need for selective inhibitors and costly methods to purify α-l-rhamnosidase or inactivate β-d-glucosidase [2,77] while maintaining high residual α-rhamnosidase activity. For this purpose, Vila-Real et al. [2] selectively inactivated β-d-glucosidase naringinase from *P. decumbens* at 81.5° C and pH 3.9, retaining 78% of α-l-rhamnosidase activity, resulting in isoquercitrin with a 61% yield.

The enzymatic bioconversion of rutin to isoquercitrin was performed using naringinase from *P. decumbens* [77]. The researchers found that the process depends on the pH of the environment and is highest at pH 6.0. At acidic pH, the activity of α-l-rhamnosidase from naringinase increased, while β-d-glucosidase showed little enzymatic activity. Due to the removal of rhamnose by α-l-rhamnosidase, more than 92% of the rutin was hydrolyzed to isoquercitrin. The solubility of isoquercitrin in water increased by 69- and 328-fold compared to rutin and quercetin, respectively. Due to its increased solubility, isoquercitrin is a more effective and bioavailable food ingredient than rutin and quercetin.

Gerstorferová et al. [79] inactivated β-d-glucosidase at pH 8.0 and 70 °C, thereby obtaining only α-l-rhamnosidase activity from *A. terreus*. Recombinant α-l-rhamnosidase was also used in the study. cDNA of rhamnosidase was cloned from *A. terreus*, sequenced, and expressed in the yeast *Pichia pastoris*. Both native and recombinant α-l-rhamnosidase efficiently catalyzed the conversion of rutin to isoquercitrin. Wang et al. [78] showed that hesperidinase, also containing α-l-rhamnosidase and β-d-glucosidase, could be used to produce isoquercitrin by selectively removing the terminal rhamnose from rutin. At pH 7.0, α-l-rhamnosidase hesperidinase showed good catalytic ability to produce isoquercitrin, while under these conditions, β-d-glucosidase lost enzymatic activity. In addition, increased conversion of rutin to isoquercitrin was noted by adding some metal ions (K^+^, Li^+^, Mg^2+^, Zn^2+^, and Al^3+^).

The antioxidant and antiproliferative potential of rutin was also studied after enzymatic hydrolysis carried out by α-l-rhamnosidase (hesperidinase from *Penicillium* sp. and naringinase from *P. decumbens)*, previously heated to 70 °C for 30 min to inactivate the undesired activity of β-d-glucosidase. The antioxidant capacity of rutin increased by about 30% after 4 h of reaction with hesperidinase, while an increase of about 10% was observed with naringinase [82].

An ethanolic extract of mulberry fruit (*Morus* spp.) has been shown to exhibit anti-allergic activity, which is attributed to the presence of bioactive compounds such as cyanidin 3-rutinoside, cyanidin 3-glucoside, and rutin. Adding naringinase to the extract enhances the inhibitory effect on the allergic response in IgE-activated mast cells, probably through the enzymatic hydrolysis of rutin to quercetin, known for its potent anti-inflammatory and antihistamine properties. Combining mulberry extract with naringinase may, therefore, find application in treating allergic disorders and as an ingredient in health-promoting functional foods [83].

### 3.5. Production of Functional Beverages with Enhanced Antioxidant Activity

The properties of grapefruit juice treated with naringinase from *P. decumbens* and adsorbed by Amberlite IRA-400 were compared [24]. In addition to reducing the bitter taste of the juice, the antioxidant capacity was studied, as well as the protective effects on lipid peroxidation, glutathione oxidation, and DNA damage. In juice subjected to both enzymatic treatment and adsorption on ion exchange resin, naringin content was reduced, making the taste of both juices acceptable to consumers. However, the antioxidant potential and free radical scavenging capacity were higher in the naringinase-treated samples. In addition, juice containing the enzyme provided more excellent protection against glutathione oxidation and lipid peroxidation than juice treated with exchange resin. Both juices were equally effective in reducing hydroxyl-radical-induced DNA damage in a dose-dependent manner. It can be concluded that, to preserve the antioxidant capacity and protect the bio-molecules of freshly squeezed grapefruit juice, the enzymatic action with naringinase was more effective than physical adsorption.

The effects of α-l-rhamnosidase, β-d-glucosidase, and their combinations on the content of naringin, polyphenols, antioxidant activity, and flavor in orange juice were studied. Both α-l-rhamnosidase and β-d-glucosidase increased the content of antioxidants, flavonoids, and polyphenols in the juice. However, a better result was obtained by using both enzymes simultaneously. The simultaneous use of α-l-rhamnosidase and β-d-glucosidase showed more excellent antioxidant activity than the use of single α-l-rhamnosidase or β-d-glucosidase. Combining both enzymes also contributed to a significant improvement in the flavor and aroma of orange juice relative to single enzymes [84].

The bioconversion of orange (*C. sinensis*) and lime (*C. latifolia*) juices by α-l-rhamnosidase and β-d-glucosidase, alone or in combination, was also investigated to hydrolyze most of the flavonoid glycosides in the juices and obtain derivatives with higher antioxidant activity. The antioxidant activity of both enzyme-reactivated juices was higher than those without enzymes. The antioxidant activity determined by the DPPH method of orange juice increased by about 30% after 4 h of reaction with naringinase. Enzyme-treated lime juice did not show higher antioxidant activity [1].

α-rhamnosidase and β-glucosidase can be used in producing Ginko tea or tea beverages to produce products with a higher content of flavonoids, ginkgolides, and aromatic components. This tea is a type of health food made from *Ginkgo biloba* (ginkgo *biloba*) leaves; however, consumers do not accept the product due to its unpleasant taste. The flavor was improved through enzymatic deglycosylation of many flavone glycosides, and bioactive compounds from ginkgo tea leaves were released. In addition, the bioconversion of flavonoids and ginkgolides to their bioactive absorptive forms was increased [50].

The health-promoting properties of polyphenols present in fruits, vegetables, and other foods such as juices, tea, and wine have been widely studied in vitro, ex vivo, and in vivo [52,56,85].

Grapefruit juice may be a potential growth medium for lactic acid bacteria. Tran et al. [9] developed and carried out a process to simultaneously remove bitterness and produce probiotic grapefruit juice by using naringinase from probiotic bacteria in simultaneous fermentation and reduction in naringin concentration. The grapefruit juice was fermented with mono and mixed probiotic cultures that showed a high capacity to produce naringinase, i.e., *L. plantarum 01*, *L. rhamnosus* B01725, *L. fermentum* D13, and *B. bifidum* B7.5 strains. It resulted in a reduction in naringin concentration by about 28% after 24 h. Imece et al. [86] added the probiotic bacteria *L. plantarum* ACC 54, *L. plantarum* ACC 28, and *L. plantarum* 250, which exhibit naringinase activity, to grapefruit juice. The tested *Lactobacillus* strains were able to reduce the concentration of naringin. After three days, an improvement in the physicochemical properties of grapefruit juice was observed. However, the long storage period caused some changes in physicochemical properties, antioxidant activity, and total phenolic content.

The lactic fermentation of fruit juice provides a “functional food” because it contains bioactive compounds such as fiber, oligosaccharides, and bacteria that promote intestinal microflora balance [9].

Pure *Clavispora lusitaniae* yeast, isolated from whey beverages, was used to make a low-alcohol, naturally carbonated, fermented, bitter grapefruit beverage. The grapefruit juice is also a medium for the naringinase-producing yeast *C. lusitaniae*. After three months of refrigerated storage, the concentration of naringin was reduced by 43%. The resulting beverage contained 0.76% alcohol [11]. Sahota and Kaur [87] used *C. lusitaniae* yeast to produce a low-alcohol, naturally carbonated, fermented beverage from kinnow. Purified naringinase from the same yeast was added to remove the bitter taste of kinnow fruit under optimized fermentation conditions (temperature 50 °C, pH 4). A similar study on kinnow juice with *C. lusitaniae* yeast, during a 3-month storage period at 4 °C, observed reductions in the concentrations of limonin and naringin of 54 and 64.8%, respectively.

The effect of naringinase on enhancing the antioxidant activity of beverages depends on their composition, particularly alcohol content, and pH, which may limit the enzyme’s effectiveness. Prolonged storage can also alter the beverages’ antioxidant activity and physicochemical properties.

### 3.6. Other Applications of Naringinase and Its Subunits

Sugar and natural sweeteners are important additives that improve the taste of food items. Natural sweeteners are in high demand due to growing health concerns about sugar consumption. α-l-rhamnosidase and β-glucosidase are used in the production of sweeteners.

β-d-glucosidase from naringinase can be used in steviol production by detaching glucose molecules from stevioside. Steviol glycosides from the leaves of *Stevia rebaudiana Bertoni* (Compositae) or *Stevia suavissimus* (Rosaceae) share the same aglycone–steviol. They differ in the content of carbohydrate residues, that is, mono-, di- and trisaccharides containing glucose and/or rhamnose at the C-13 and C-19 positions. These structural differences give the glycosides their relative sweetness and flavor quality. The main glycosides are stevioside, rubusoside, steviol monoglucoside, steviol monoglucosyl ester, and steviolbioside. Steviol is an important aglycone of steviol glucosides, providing an alternative to sucrose, and is also used as a pharmaceutical compound to improve cognitive functions such as learning, memory, alertness, and psychotic stability, as well as a plant growth factor. These compounds also have immunomodulatory properties and protect against hyperglycemia, hypertension, inflammation, cancer, and diarrhea [88].

β-glucosidase has hydrolyzing activity against stevioside, rubusoside, steviol monoglucoside, and steviol monoglucosyl ester. The β-glucosidase can hydrolyze steviosides to rubusoside, steviolbioside, and steviol monoglucoside, and then to steviol [89]. Purified β-glucosidase naringinase from *P. decumbens* was used to hydrolyze steviol glycosides. The enzyme produced steviol by hydrolyzing rubusoside and steviol monoglucoside. The yield for obtaining steviol using β-glucosidase is 64% from a 47 mM stevioside solution at 55 °C and pH 4.0 [88]. Using naringinase from *Penicillium* sp., Nguyen et al. [90] converted stevioside to rubusoside with a yield of about 51%. Rubusoside is a sweetener and a solubilizing agent what can be used in the pharmaceutical industry [44].

α-l-rhamnosidase is also used in the production of sweeteners. Through a combination of hydrogenation and enzymatic hydrolysis reactions, α-l-rhamnosidase can produce the natural sweetener dihydrochalcone glycoside trilobatin. In this method, naringin isolated from citrus waste was hydrogenated to dihydrochalcone, from which the rhamnose molecule was then detached using immobilized α-l-rhamnosidase as a catalyst [91].

Beverages containing one or more steviol glycosides, such as rebaudioside A, rebaudioside D, and rebaudioside M, may exhibit an insufficiently sweet taste compared to the initial sweetness in sugar-sweetened beverages. Naringenin, an aglycone of naringin, can be added to beverages to improve the perception of sweet taste. Naringenin can be used as a beverage additive or an ingredient in a sweetener composition to adjust the sweetness attributes of beverages [92]. EFSA (European Food Safety Authority) approved the use of naringenin as a flavoring in food [93].

The production of natural sweeteners other than sucrose requires using one of the enzymes present in naringinase, which necessitates the development of an effective method for the selective inactivation of the other enzyme. Research in this area has emerged relatively recently and still requires further intensive investigation.

Tilianin, a rare flavonol glycoside, is gaining increasing attention for its various biological properties, including sedative, antihypertensive, and anticonvulsant effects. However, it is relatively expensive and difficult to obtain due to its low content in plants. A selective and efficient linear biotransformation process has been developed to produce thylacine by naringinase. Linarin contains a rutinoside attached to the C-7 site of acacetin and can be converted to tilianin (acacetin-7-*O*-β-d-glucoside) by selective hydrolysis of the rhamnosyl group by naringinase. It has been shown that temperature can modulate the activity of α-l-rhamnosidase and β-d-glucosidase from naringinase. Under optimized conditions (pH 7, 60 °C), linarin was almost wholly converted to thylanine (98.9%) [14,15,94]. Using naringinase to produce tilianin requires neutral pH and elevated temperature, which are often not optimal conditions for the enzyme’s activity.

In addition, naringinase and hesperidinase can aid in extracting proteins from grapefruit seeds remaining after cold-pressing the oil. Incubation of crushed seeds with these enzymes prior to protein extraction leads to a reduction in viscosity, which may facilitate protein extraction. These results suggest potential applications of grapefruit seed proteins in the food industry as food enrichment ingredients, animal nutrition, and other industrial applications [85].

The action of naringinase can affect the chemical and physical properties of mesocarp powder from the bitter fruit of *Borassus flabellifer*. Naringinase-reduced mesocarp powder has good water retention, swelling, and wettability. Naringinase reduced the particle size of the mesocarp by 33.2%, causing an increase in its surface area. The larger surface area retains more water/oil molecules, contributing to higher water/oil capacity. However, the mesocarp powder’s solubility, swelling, and wettability were markedly reduced after naringinase treatment. Due to naringinase-mediated deagglomeration, *Borassus flabellifermma* mesocarp powder has the potential as a functional ingredient to increase fiber content in products that require hydration, low calorie content, and high fiber content, such as pasta, energy bars, and breakfast cereals [95].

Naringinase can also be used to produce soy-based foods to enrich them with free isoflavones. Isoflavones are polyphenolic molecules present in soybeans that have antioxidant and phytoestrogenic properties. Some isoflavones are present in the form of a free aglycone (e.g., daidzein, genistein, and glycitin) or their respective glucoside conjugate (e.g., daidzein, genistein and glycitin). In soybeans, isoflavones are mainly found in the glucoside-bound form. However, isoflavones in aglycone form show higher bioavailability [44]. Soy extracts were biotransformed with tannase from *Paecilomyces variotii* and immobilized β-glucosidase from naringinase from *P. decumbens*. As a result of the enzymatic processes, there were significant changes in the profiles of isoflavones in the soy beverage due to the bioconversion of glycosidic forms (daidzin and genistein) into aglycone forms (daidzein and genistein) [96].

## 4. Conclusions

In conclusion, this review underscores the importance of naringinase in the food industry, and the information available in scientific publications can significantly help guide the application of this enzyme. Naringinase is widely used to improve the quality of food products because it can hydrolyze compounds linked to rhamnose or glucose. The most talked about use of naringinase is to improve flavor and aroma in the juice and wine industries. The proposed solutions in this area, using free and immobilized enzymes, indicate their potential industrial application. This review also presents other potential applications of naringinase in food technology. Naringinase, selectively hydrolyzing naringin, hesperidin, and rutin, contributes to the increased bioavailability of flavonoids. From the perspective of selective flavonoid hydrolysis, it is essential to develop conditions that allow effort the effective deactivation of β-d-glucosidase while maintaining maximum activity of α-l-rhamnosidase, the second enzyme in the naringinase complex. Deglycosylation of flavonoids solely by α-l-rhamnosidase, resulting in glucoside-containing compounds, can enhance their bioavailability. Therefore, inhibition of β-d-glucosidase from naringinase is crucial for improving the absorption of flavonoid glycosides. Additionally, β-glucosidase catalyzes the hydrolysis of polysaccharides into simple sugars, which increases blood glucose levels. Inhibiting their activity slows carbohydrate digestion and may be beneficial in managing metabolic disorders such as type 2 diabetes—one of the significant health challenges of the 21st century. Ideally, β-d-glucosidase inhibitors should be of natural origin, with promising candidates including hibiscus or *Hericium erinaceus* (lion’s mane). Inhibition can also be achieved by adjusting reaction conditions to favor high α-l-rhamnosidase activity with minimal β-d-glucosidase activity. Moreover, enhancing α-l-rhamnosidase activity through stabilization at elevated temperatures may further support targeted flavonoid deglycosylation. Another approach may be to develop, using genetic engineering methods, strains of microorganisms synthesizing naringinase with high α-l-rhamnosidase activity and low β-d-glucosidase activity.

Hydrolyzing polyphenols increases the antioxidant capacity of products. It can also produce functional beverages with increased amounts of bioactive flavonoids. α-l-rhamnosidase and β-glucosidase from naringinase are used to produce sweeteners and can be used in the soy products industry.

The presented applications of the enzyme result from the ability of naringinase to deglycosylate the compounds present in food. Deglycosylation of other, as-yet-unexplored substrates of naringinase may lead to bioactive compounds with new industrial applications.

## Figures and Tables

**Table 1:** Debittering of citrus juices using naringinase.

Form of Enzyme Used	Source of Enzyme	Product	Condition of Juice Debittering	Reduction in Naringin	References
Naringinase immobilized in calcium alginate bead	*P. decumbens*	Orange juice	160 MPa, 37 °C, 20 min 500 enzyme mg·dm^−3^ juice)	75%	[22]
Naringinase entrapped in *k*-carrageenan beads	*P. decumbens*	Grapefruit juice	30 °C, 120 min, 4 (juice): 1 (*k*-carrageenan beads enzyme)	70%	[25]
Free naringinase	*P. decumbens*	Grapefruit juice	20 °C, 24 h, 25 cm^3^ juice, naringinase (0.4 U·cm^−3^),	46.8%	[24]
Naringinase immobilized on mesoporous silica	-	White grapefruit juice	60 °C, 30 min	95.03%	[26]
Free naringinase	*A. niger*	Honey pomelo juice	40 °C, 60 min	about 90%	[5]
Free naringinase	*P. purpurogenum*	Grapefruit juice	40 °C, 4 h 100 U of enzyme/100 cm^3^ juice	74%	[7]
Free naringinase	*A. oryzae*	Pomelo juice	45 °C, 60 min	approximately 99%	[6]
Free naringinase	-	“Bibila sweet” oranges	50 °C, 4 h 1.0 g of enzyme/dm^3^ juice	86%	[27]
Naringinase immobilized on electrospun cellulose acetate nanofibers	-	Grapefruit juice	-	22.72%	[28]
Free naringinase	*C. albidus*	Grapefruit juice	40 °C and 60 °C, 60 min	84% and 100%	[8]
Naringinase immobilized on chitosan microspheres activated with glutaraldehyde	*A. niger*	Grapefruit juice	40 °C, 5 h	75%	[29]
Naringinase encapsulated in nano-chitosan and nano-alginate	*T. longibrachiatum*	Grapefruit juice	50 °C, 60 min	50.5% and 44.15%	[30]
Naringinase immobilized on polyethersulfone ultrafiltration membrane	*P. decumbens*	Grapefruit juice	45–50 °C 0.025 MPa	50.1 ± 0.3%	[31]
Free naringinase	*Thermomicrobia* sp.	Kinnow juice pomace	50 °C, pH 4.5, 4 h,	65.95%	[32]
Free naringinase	*A. niger*	Kinnow juice	Room temperature, 12 h	40.0%	[33]
Naringinase immobilized in agarose supports	*A. aculeatus/A. niger*	Grapefruit juice	30 °C, 24 h	74%	[23]
Free naringinase	*B. amyloliquefaciens*	Grape juice	37 °C, 20 min	23.4%	[10]
Free naringinase	*B. subtilis*	Lemon, grapefruit, orange, and mandarin	4 h, 40–50 °C	33–36%	[4]
Naringinase adsorbed onto a macroporous resin	*A. niger*	Pomelo juice	60 °C, 160 min	53.06%	[34]
Free naringinase	-	Grapefruit juice	35 °C, 3 h 50 min	55.77%	[35]
Free naringinase	*Serratia marcescens*	Grapefruit juice	55 °C, 90 min	85.93%	[36]
Naringinase chemically aminated prior to its immobilization on glyoxyl-agarose	*A. aculeatus/A. niger*	Grapefruit juice	30 °C, 24 h	74%	[37]
Naringinase immobilizes onto polydopamine-coated magnetic iron oxide nanoparticles	*A. aculeatus/A. niger*	Grapefruit juice	50 °C, 24 h	56%	[38]
Free naringinase	*Bacillus megaterium*	Lemon and tangerine juice	37 °C	45.78% (lemon juice) 42.71% (tangerine juice)	[39]

**Table 2:** The use of naringinase for the hydrolysis of hesperidin.

Form of Enzyme	Source of Enzyme	Product	Condition of Juice Debittering	Results	References
Free naringinase	*A. sojae*	Orange juice/orange peel	37 °C, 24 h, enzyme solution: 1.7–2 mg/mL	Production yield of Hes-7-G * was 71% for orange juice and 78% for orange peel	[56]
α-l-rhamnosidase and β-d-glucosidase	*A. niger*	Reaction mixture (20 mg of hesperidin and 40 mg of freeze-dried whole-cell catalysts was mixed with 2 cm^3^ of acetate buffer)	pH 5.0, 40 °C, 24 h	93.9 ± 1.4% conversion of hesperidin; 73.3 ± 9.2% Hes-7-G; 26.7 ± 9.2% hesperetin	[53]
β-glucosidase	*Pyrococcus furiosus*	Orange peel extract	95 °C for 12 h (pH 5.5) 100% citrus extract, 0.85 U cm^−3^ enzyme	100% (*w*/*v*) conversion of hesperidin to 9.0 g·dm^−3^ hesperetin after 9 h, with a productivity of 1.00 g·dm^−3^·h^−1^	[73]
α-l-rhamnosidase	*A. niger*	Reaction mixture (1 cm^3^ 0.5 mM hesperidin, 0.98 cm^3^ phosphate citrate, and 20 mm^−3^ α-l-rhamnosidase)	0.7 M sorbitol 60 °C, pH 4.5, 10 min	63.26% hesperidin was hydrolyzed to Hes-7-G. completely hydrolyzed after 10 h reaction	[55]
Naringinase and β-cyclodextrin		β-cyclodextrin content 57.5%, hesperidinase, naringinase	70 °C, pH 4.5	98%	[74]

* Hes-7-G—hesperetin 7-*O*-glucoside.

**Table 3:** The use of naringinase for the hydrolysis of rutin.

Form of Enzyme	Source of Enzyme	Reaction Mixture	Condition of Reaction	Results	References
Free naringinase (inactivation of β-d-glucosidase activity)	*P. decumbens*	5 mM rutin, 50 mg·dm^−3^ enzyme	Residual activity of α-l-rhamnosidase (78%), pH 3.4, 60.0 °C, 6 h	Production yield of isoquercitrin, 61%	[2]
Free naringinase (inactivation of β-d-glucosidase activity)	*P. decumbens*	5 mM rutin, 50 mg·dm^−3^ enzyme	pH 3.4, 60.0 °C, 6 h	Production yield of quercetin, 86%	[2]
Free naringinase (inactivate the unwanted β-d-glucosidase)	*A. terreus*	100 g·dm^−3^ rutin, 20 cm^3^ enzyme	pH 8.0, 70 °C, 24 h	Production yield of isoquercitrin, 61%; volumetric productivity (up to 300 g·dm^−3^)	[79]
Hesperidinase (contains both α-l-rhamnosidase and β-d-glucosidase activities; inactivation of β-d-glucosidase activity)	*A. niger*	20 cm^3^ saturated solution of rutin, 10 mg enzymes	pH 7.0, 40 °C, 30 h	Production yield of isoquercitrin 50.06%	[78]
Free naringinase (high α-l-rhamnosidase activity, very low β-d-glucosidase activity)	*P. decumbens*	1.5 mM rutin, 0.1 mg·dcm^−3^ enzyme	pH 6, 37 °C, 12 h	Production yield of isoquercitrin, 92%	[77]
Free naringinase	*P. decumbens*	0.8 g·dm^−3^, 3000 U·dm^−3^ enzyme	40 °C, 20 min, ultrasound irradiation	Production yield of isoquercitrin, 95.20 ± 2.52%	[75]
Graphene-immobilized naringinase flowing in microchannels	*P. decumbens*	0.05 g·dm^−3^ rutin, 8 μL, min^−1^ flow rate	40 °C, 10 min	92.24 ± 3.26% isoquercitrin	[76]
Photo pattern-immobilized naringinase on a microchip	*P. decumbens*	0.03 g·dm^−3^ rutin, 5 µL, min^−1^ flow rate	45 °C, 5 min	93.28 ± 1.12% conversion of rutin, 87.98 ± 1.1% isoquercitrin yield	[80]
Free α-l-rhamnosidase and β-d-glucosidase	*A. niger*	20 mg rutin, 40 mg of freeze-dried whole-cell catalysts	pH 5.0, 40 °C, 24 h	97.2 ± 0.9% conversion of rutin; 94.2 ± 1.6% isoquercitrin; 5.86% ± 1.6% quercetin	[53]

## Data Availability

No data were used for the research described in the article.

## Supplementary Materials

The following supporting information can be downloaded at: https://www.mdpi.com/article/10.3390/molecules30112376/s1.

## Abbreviation

The following abbreviations are used in this manuscript:

Hes-7-G	hesperetin 7-O-glucoside
